# Efficacy and Safety of Enoxaparin versus New Oral Anticoagulants to Prevent Venous Thromboembolism after Total Hip Replacement: A Systematic Review and Meta-Analysis

**DOI:** 10.3390/jpm12010107

**Published:** 2022-01-14

**Authors:** Mohammed Farhan A Alfarhan

**Affiliations:** Department of Surgery, Division of Orthopedics, College of Medicine, King Faisal University, Al-Ahsa 31982, Saudi Arabia; malfarhan@kfu.edu.sa; Tel.: +966-135898989

**Keywords:** deep venous thrombosis, enoxaparin, oral anticoagulants, total hip replacement, pulmonary embolism, venous thromboembolism

## Abstract

Prophylactic anticoagulant therapy is recommended for reducing the risk of venous thromboembolism (VTE) after a total hip replacement (THR). However, it is not clear which anticoagulant is preferable. Hence, a systematic review and meta-analysis of randomized double-blind controlled trials (RDBCTs) were conducted to investigate the clinical efficacy and safety of enoxaparin in comparison with newer oral anticoagulants for the prevention of VTE after THR. The Cochrane Library, Scopus, Web of Science, Embase, and PubMed/Medline databases were used for PICO search strategy. Relative risks (RR) of symptomatic VTE, clinically relevant bleeding, mortality, and a net clinical endpoint were estimated employing a random effect meta-analysis. ITC and RevMan software were used for indirect and direct comparisons, respectively. Nine RDBCTs comprising 24,584 patients were included. As compared to enoxaparin, a reduced risk for symptomatic VTE was observed with rivaroxaban (confidence interval [CI]: 0.32–0.77; RR: 0.46%) and comparable with apixaban (0.12–1.26; 0.42%) and dabigatran (0.22–2.20; 0.70%). Contrarily to enoxaparin, a greater risk for clinically relevant bleeding was observed with rivaroxaban (1.03–1.48; 1.23%), comparable with dabigatran (0.96–1.33; 1.10%) and reduced with apixaban (0.19–5.66; 0.96%). In indirect or direct comparisons, the interventions did not differ on the net clinical endpoint. In conclusion, the findings of this meta-analysis revealed no significant difference in the efficacy and safety of new oral anticoagulants as compared to enoxaparin for the prevention of VTE after total hip replacement surgery.

## 1. Introduction

Total hip replacement (THR) is an effective and common treatment for degenerative joint diseases including osteoarthritis [1]. Venous thromboembolism (VTE) (i.e., pulmonary embolism [PE] and deep venous thrombosis [DVT]) is a vital reason for long-term morbidity, has significant healthcare costs, and represents a preventable cause of death [2]. Every patient undergoing joint replacement surgery is at risk of VTE due to decrease in perioperative mobility and duration of surgery. Hence, all such patients receive around 35 days of anticoagulation therapy after surgery for reducing the risk of VTE [3]. At 3 months, the rate of VTE after THR is variable (i.e., nearly 2% for PE and around 5% for DVT) among anticoagulated patients [4]. 

Anticoagulants to prevent VTE include newer oral agents (i.e., dabigatran [Pradaxa; Boehringer Ingelheim International, Germany] [5], apixaban [Eliquis; Bristol-Myers Squibb/Pfizer EEIG, United Kingdom] [6], and rivaroxaban [Xarelto; Bayer Pharma, Germany]) [7], injectable agents (i.e., low molecular weight heparin [LMWH]), and simple oral agents (i.e., aspirin). Aspirin, having a remarkable safety profile, is well tolerated, is easily administered, is inexpensive, and requires no blood monitoring [8]. Presently, aspirin is utilized off-label to prevent VTE in both the United Kingdom and the United States. However, some concerns related to the newer and more expensive anticoagulants exist, including greater bleeding risks and wound complications [8]. Hence, significant debate exists regarding which drugs should preferably be administered to balance the clinical efficacy against cost and bleeding risks.

Several organizations, including the United Kingdom National Institute for Health and Care Excellence (NICE), the American College of Chest Physicians (ACCP), and the American Academy of Orthopaedic Surgeons (AAOS), have made major efforts to formulate guidelines for the prevention of VTE that utilize a strict approach to synthesize evidence and recommendations [9,10,11]. According to the AAOS (2011) guideline, which was based on a medium evidence level, all patients undergoing total knee replacement (TKR) or THR should receive VTE prophylaxis (mechanical and/or pharmacological) [9]. Nonetheless, at that time, the AAOS could not recommend for or against any particular VTE prophylactic drugs due to lack of evidence [9]. In 2012, aspirin was endorsed by the ACCP for the prophylaxis of VTE after TKR or THR, with a medium level of evidence (i.e., IB Grade) in comparison with no VTE prophylaxis, which is a similar evidence level designated to both newer oral and injectable drugs in comparison with no VTE prophylaxis [10]. In 2018, aspirin alone was recommended by the NICE as an option for the prevention of VTE after TKR; however, after THR 10 days of LMWH is required by the patients prior to receiving aspirin, or they might only receive LMWH or the newer and more expensive oral drugs [11]. 

In Europe, the most frequently used anticoagulants for the prophylaxis of VTE are LMWH, including enoxaparin [12]. Enoxaparin is administered via subcutaneous injection. According to the multivariate analysis of findings from a Spanish report, chronic lung disease and receiving thromboprophylaxis with LMWH for <3 weeks were identified as the only two determinants that were independently correlated with a greater risk for VTE [12]. Many studies have reported enoxaparin to be cost-effective as compared to unfractionated heparin, warfarin, and LMWH as prophylaxis after orthopedic surgery [13]. Although enoxaparin might represent a cost-effective option, the debate remains whether it must be a preferable option in comparison with newer oral anticoagulants in terms of efficacy and safety for VTE prophylaxis. Owing to the prevalence of VTE, the need for evidence of which agent is useful to prevent this post-THR complication is imperative. Hence, the current systematic review and meta-analysis aimed to investigate the clinical efficacy and safety of enoxaparin in comparison with newer oral anticoagulants for the prevention of VTE after THR.

## 2. Results

### 2.1. Study Identification 

**Figure 1:**
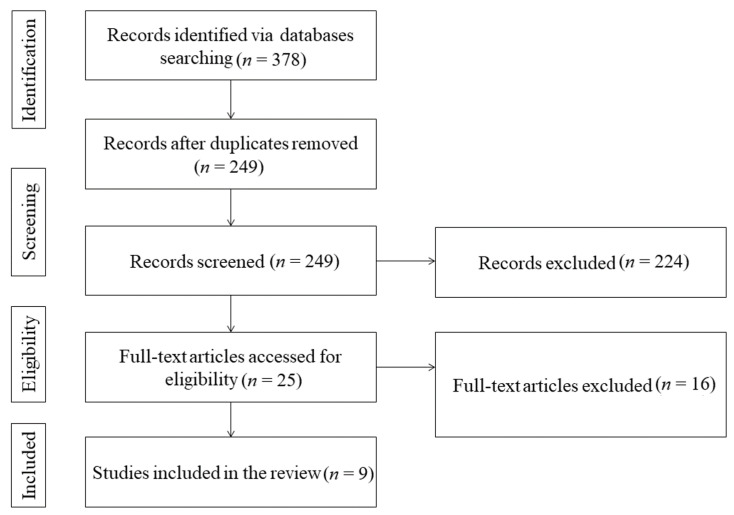
PRISMA flow diagram.

Figure 1 depicts the method of article screening and selection. The primary search yielded 378 potentially relevant articles. After removing the duplicates employing manual confirmation and Endnote software, 249 articles remained. The 224 articles were excluded since they did not meet the inclusion criteria. After screening the publication title and abstract, only 25 publications met the eligibility criteria. The 16 articles were discarded after the verification of the full text of the remaining 25 articles. Finally, 9 studies that satisfied all the inclusion criteria were included in the present systematic review [14,15,16,17,18,19,20,21,22]. 

### 2.2. Study Characteristics

Table 1 summarizes the main characteristics of the 9 included studies. A total of 24,584 patients (10,941 received enoxaparin; 5475 received dabigatran; 5137 received rivaroxaban; and 2708 received apixaban) participated in the included studies, 55.58% (13,663) of whom were women. Interestingly, all the included studies were from Europe (Sweden [14,15,16,17,18,21,22], the United Kingdom [19], and Denmark [20]). The age of the participants ranged between 18 and 93 years, with a mean age of 63.2 years. The most frequent comparators were rivaroxaban [14,15,16,18,19], dabigatran [17,21,22], and apixaban [20]. VTE, DVT, and PE were reported by all the included studies.

### 2.3. Primary Efficacy Outcome Analysis 

**Figure 2:**
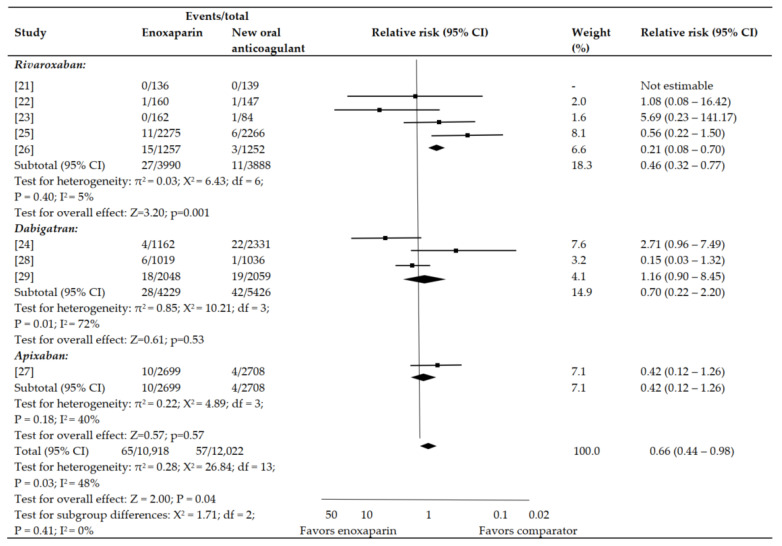
Symptomatic VTE.

Rivaroxaban was linked with a decrease in risk for symptomatic VTE as compared to enoxaparin (*p* = 0.001; 95% CI: 0.32–0.77; RR: 0.46%) (Figure 2). In comparison with enoxaparin, neither apixaban (*p* = 0.18; 0.12–1.26; 0.42%) nor dabigatran (*p* = 0.01; 0.22–2.20; 0.70%) decreased the risk for symptomatic VTE (Figure 2).

No statistical heterogeneity was observed among the included articles for symptomatic VTE that compared enoxaparin with apixaban, dabigatran, or rivaroxaban. However, evidence of statistically significant heterogeneity was noticed for symptomatic VTE in the studies including dabigatran (*p* < 0.01; I^2^ = 72%) (Figure 2). No reason for heterogeneity was determined after examining the presence of an outlier publication, regimen of enoxaparin, or daily dosage of dabigatran. The influence on symptomatic VTE in comparison with enoxaparin was comparable to daily dabigatran dosages of 150 mg (*p* = 0.71; 0.29–2.13; 0.78%) and 220 mg (0.59; 0.21–2.67; 0.65%). 

After the inclusion of symptomatic VTE events that happened amidst follow-up duration, the outcomes were comparable to those of the primary analysis; apixaban (*p* = 0.41; 0.33–1.48; 0.70%), dabigatran (*p* = 0.67; 0.40–1.73; 0.89%), and rivaroxaban (*p* < 0.05; 0.31–0.81; 0.59%) in comparison with enoxaparin. 

### 2.4. Secondary Efficacy Outcome Analysis 

Rivaroxaban was linked with a considerable decrease in risk for symptomatic DVT as compared to enoxaparin (*p* < 0.05; 0.24–0.69; 0.42%), while a non-significant trend was observed for symptomatic PE (*p* = 0.78; 0.34–2.53; 0.87%). The risk of total VTE or all cause fatality (*p* < 0.05; 0.40–0.82; 0.54%) together with major VTE or VTE-related mortality (*p* < 0.05; 0.19–0.80; 0.39%) was also reduced by rivaroxaban. 

There was no association between a varying risk for symptomatic PE (*p* = 0.29; 0.32–1.45; 0.67%) or DVT (*p* = 0.79; 0.21–3.61; 0.84%) and rivaroxaban in comparison with enoxaparin. Dabigatran was linked with a greater risk for total VTE or all cause fatality as compared to enoxaparin (*p* = 0.33; 0.90–1.15; 1.02%) and a comparable risk for major VTE or VTE-related mortality (*p* = 0.51; 0.61–1.52; 0.91%). The risk for total VTE or all cause fatality was comparable between enoxaparin and 220 mg dabigatran (*p* = 0.92; 0.81–1.06; 1.01%), while a greater risk was observed using 150 mg dabigatran as compared with enoxaparin (0.51; 0.99–1.40; 1.12%). Regarding major VTE or VTE-related fatality, no significant difference was observed between enoxaparin and 220 mg dabigatran (*p* = 0.28; 0.49–1.10; 0.81%) or between enoxaparin and 150 mg dabigatran (*p* = 0.48; 0.79–1.58; 1.08%). 

### 2.5. Primary Safety Outcome Analysis

**Figure 3:**
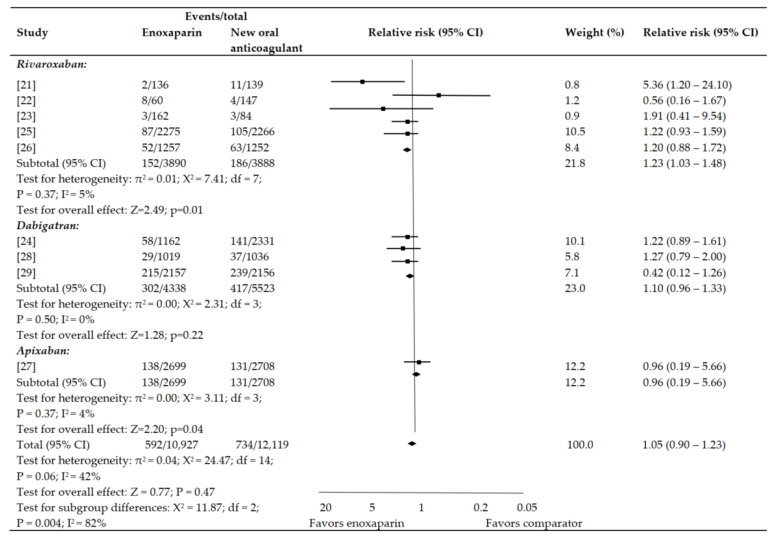
Clinically relevant bleeding.

Regarding increase in risk for clinically relevant bleeding, a non-significant trend was observed by rivaroxaban (*p* = 0.37; 1.03–1.48; 1.23%) and dabigatran (*p* = 0.50; 0.96–1.33; 1.10%) as compared to enoxaparin (Figure 3). A comparable risk was observed with enoxaparin as compared to 150 mg (*p* = 0.43; 0.86–1.39; 1.20%) and 220 mg dabigatran (*p* = 0.30; 0.96–1.28; 1.20%). Contrarily, a considerably decreased risk for clinically relevant bleeding was associated with apixaban as compared to enoxaparin (*p* = 0.37; 0.19–5.66; 0.96%). No statistical heterogeneity was observed among the included articles for this outcome that compared enoxaparin with apixaban, dabigatran, or rivaroxaban (Figure 3). 

### 2.6. Secondary Safety Outcome Analysis 

A non-significant trend towards a greater risk for major bleeding was associated with rivaroxaban as compared with enoxaparin. In contrast to enoxaparin, dabigatran was linked with a comparable risk for major bleeding (*p* = 0.68; 0.61–1.48; 0.98%) and a non-significant trend towards a greater risk for clinically relevant minor bleeding (*p* = 0.14; 0.99–1.46; 1.21%). A non-significant trend towards a reduced risk for major bleeding was associated with apixaban as compared to enoxaparin (*p* = 0.52; 0.49–1.34; 0.80%), which was within the limits of statistically significant difference for clinically relevant minor bleeding (*p* = 0.05; 0.72–0.99; 0.81%). There were no significant trends observed in the risk for mortality between enoxaparin and the newer anticoagulants. 

### 2.7. Net Clinical Endpoint

**Figure 4:**
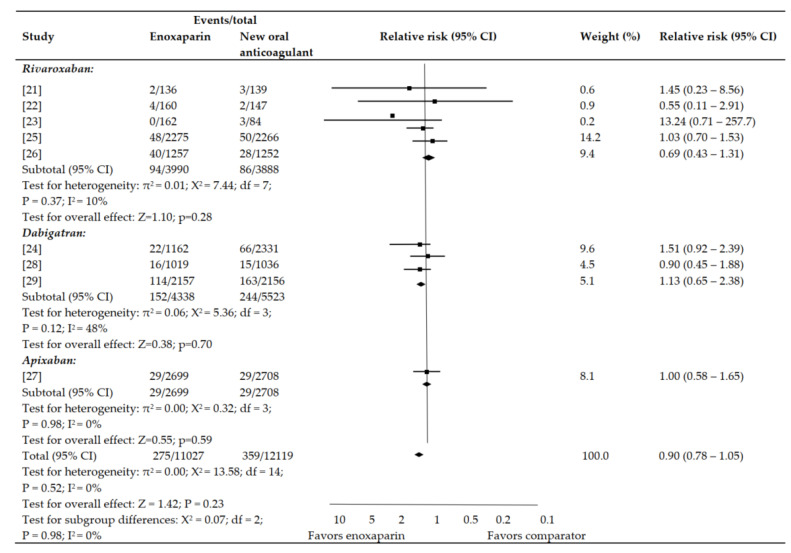
Net clinical endpoint.

There were no statistically significant differences between enoxaparin and the new oral anticoagulants on the net clinical endpoint (symptomatic VTE, major bleeding, and mortality) (Figure 4). No evidence of statistical heterogeneity was observed among the included studies. 

### 2.8. Risk of Bias

**Figure 5:**
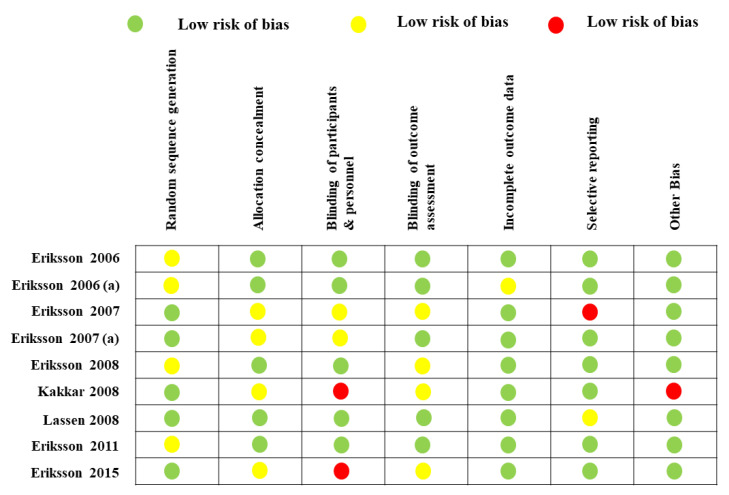
Risk of bias in the included studies.

Owing to poor reporting, it was not easy to evaluate the risk of bias of the included studies (Figure 5). A high risk of bias was observed for blinding since blinding was carried out rarely and was complicated to maintain as enoxaparin was administered subcutaneously. An effective blinding procedure was performed by five studies [14,15,18,20,21]. 

### 2.9. Indirect Comparisons

Reduced risk for VTE was associated with rivaroxaban, while apixaban appeared to be associated with the least risk for clinically relevant bleeding (Table 2). No significant differences were observed between interventions on the net clinical outcomes. 

### 2.10. Absolute Difference in Events per One Thousand Subjects

The amount of VTE avoided per one thousand subjects treated with rivaroxaban versus apixaban, dabigatran, or enoxaparin was usually comparable to those of the major bleeds (Table 3). No significant differences were observed between interventions on the net clinical outcomes.

## 3. Discussion

The present systematic review and meta-analysis aimed to assess the clinical efficacy and safety of enoxaparin in comparison with newer oral anticoagulants for the prevention of venous thromboembolism (VTE) (including deep venous thrombosis [DVT] and pulmonary embolism [PE]) after a total hip replacement (THR). The findings of the present review revealed that a greater efficacy of the newer oral anticoagulants is usually correlated with a greater tendency of bleeding in patients undergoing THR. The varying oral anticoagulants, at the point of balancing safety (major bleeding events and mortality) and efficacy (symptomatic VTE), did not differ significantly. 

According to the American Heart Association, PE results in 10,000 fatalities, and around 2 million Americans suffer from DVT every year [23]. THR has been a strong predisposing factor (odds ratio [OR] > 10) to develop VTE [24]. The evidence-based guidelines as per the ACCP recommend extensive prophylaxis for preventing VTE among patients undergoing THR [25]. Usually, it is required for such patients to continue using anticoagulants after getting discharged from the hospital; however, short durations of present-day stays in the hospital mostly cause a reduced quantity of patients taking enough prophylaxis proposed by the clinical guidelines [26]. VTE is reported to be experienced by 40–70% of subjects undergoing THR who do not take adequate prophylaxis and generally results in serious sequelae [27]. Over the past 20 years, prophylaxis of VTE has been extensively acknowledged as a worthy and efficacious strategy, and the recommendations proposed by the ACCP are another milestone in the pathway toward standard implementation of VTE prevention. Presently, subcutaneous injection of LMWH is extensively utilized in the US and Europe; a 40 mg daily dose of enoxaparin is the standard recommendation, and few randomized controlled trials have yielded valid evidence in its favor [28,29]. Although, enoxaparin has benefits over warfarin and heparin regarding the onset of anti-thrombotic action, peak time, and half-life; however, rare yet serious adverse complications including osteoporosis, thrombocytopenia and bleeding require to be considered, together with inconvenience of administration [23,30]. Hence, the development of new oral anticoagulants has been strongly encouraged [31]. 

Previous meta-analyses of apixaban, dabigatran, and rivaroxaban [32,33], rivaroxaban [34,35], apixaban [36], dabigatran and rivaroxaban [37], and dabigatran [38] have revealed segmental comparative outcomes of the many trials using these new oral anticoagulants. In these meta-analyses, somewhat varying inclusion criteria and statistical analyses were applied such as RR or OR with random or fixed effects calculations. Moreover, statistical heterogeneity was not always taken into account in the formulated findings, and hence, panoramic comparisons were not accurate. Pooled analyses were carried out for dabigatran [39] and rivaroxaban [40]; however, the included populations were not standard protocol, since in the original studies, and might in future studies, in the presence of statistical heterogeneity, unintentionally result in non-substantial outcomes [41]. To the author’s knowledge, no systematic review and meta-analysis of randomized double-blind controlled trials (RDBCTs) has been performed that summarized all new oral anticoagulants. Nonetheless, all comparisons of new oral anticoagulants were indirect, and hence, direct comparison reports will be crucial in the future. The large observational registries’ comparisons showed variations among real-life subject populations, which might hinder the estimation of clinical study data to clinical application. Variations in clinical heterogeneity and endpoint definitions in a group of comparisons also hamper sufficient indirect comparisons of some oral anticoagulant drugs [42]. 

In the present review, rivaroxaban appeared more efficacious to prevent symptomatic VTE as compared to enoxaparin, however, at the cost of a greater trend of clinically relevant bleeding. The consistent outcomes were observed throughout different included studies, without the evidence of statistical heterogeneity. 

Dabigatran appeared as efficacious as enoxaparin in reducing the risk for symptomatic VTE; however, the outcomes are worth observing by wide confidence interval and heterogeneity. According to the surrogate venographic data on total and major VTE suggested that 200 mg (high dosage) of dabigatran is persistently comparable to enoxaparin. The 150 mg (low dosage) dabigatran might be an alternative in subjects having anticipated increased dabigatran exposure [43], including those patients who suffer from moderate renal impairment and those who are >75 years old [5]. The findings of the present review revealed that the risk for clinically relevant bleeding did not differ significantly between enoxaparin and dabigatran. However, the upper limit of the 95% CI suggested that an RR of clinically relevant bleeding using dabigatran versus enoxaparin by 35% cannot be excluded. 

Apixaban was linked with a reduced risk for clinically relevant bleeding as compared with enoxaparin, however, it was associated with an increased risk for PE. Symptomatic PE takes place later in THR as compared to TKR [44,45], which may theoretically lead to an increased risk for early PE if the first dose of apixaban is delayed. Surgeons might take into account the potential advantages of earlier anticoagulation for VTE prophylaxis along with the risks for post-surgical bleeding to decide when to give the anticoagulant within the approved time frame (12–24 h post-surgery for apixaban) [7]. 

The findings of the present systematic review and meta-analysis revealed that the definition of major bleeding might exert a considerable influence on the apparent safety of the oral anticoagulant agent and that even difficult to identify alterations in the definitions might cause varying conclusions in the risk-benefit balance. 

The potential strengths of the present systematic review and meta-analysis are important to recognize. This review represents data from approximately 25,000 patients enrolled in 9 RDBCTs, and all including blinded and independent outcome assessors. The included articles were published between 2006 and 2015 and there was a lack of evidence of publication bias. In the near future, it seems improbable that an RDBCT comparing two new oral anticoagulants in THR would be performed. Hence, the outcomes of the present review yield a valuable estimate of expected relative variations on clinically relevant events between apixaban, dabigatran, and rivaroxaban in THR. There is no systematic review and meta-analysis that have been performed after 2012 [42] and 2013 [43] that summarized all new oral anticoagulant trials in a unique meta-analysis.

There are a few published studies that indirectly compared rivaroxaban with dabigatran [46,47]. The rates of symptomatic VTE were indirectly compared in only one of these studies [46]; however, two RENOVATE II trials were not included in this report, which was published later in 2011 [21] and 2015 [22], respectively.

Globally, for end-stage osteoarthritis, one of the most frequently performed elective orthopedic procedures is total joint replacement [48]. The terminology “clinical effectiveness” refers to ensuring that healthcare practice is based on the best available evidence and data of effectiveness. According to the NICE, the “clinical effectiveness of an intervention” refers to how advantageous the intervention is under every day or usual circumstances, as compared to opting for another type of care or nothing [49]. Concerns have been raised that several orthopedic surgical treatments and prostheses utilized in these treatments do not have high quality or readily available evidence regarding their clinical effectiveness for supporting their utility [50,51]. A recent systematic review reported that 24% of all hip replacement implants available to surgeons in the United Kingdom did not have evidence for their clinical effectiveness [52]. Although clinical guidelines, referred to as “systematically formulated statements for assisting surgeons and patients’ decisions regarding proper health care for particular clinical conditions” [53] aim to be disseminated by the best available evidence, they have mostly been criticized for their lack of methodological applicability [54,55]. 

Some considerations are essential while translating the outcomes from these RDBCTs into clinical practice. It is anticipated, in absolute terms, that subjects in standard clinical setup might experience a greater risk for bleeding and symptomatic VTE as compared with those included in RDBCTs, owing to the variations in personal features as well as by the exclusion criteria applied in RDBCTs (i.e., bleeding history, strong CYP3A4 inhibitors, adjuvant intervention using NSAIDs having long half-life, chronic intake of vitamin K antagonists, and severe hepatic or renal insufficiency) [56]. Importantly, aging is associated with a greater tendency for bleeding as compared to the risk for symptomatic VTE [57]. Hence, one of the primary improbabilities regarding the utilization of new oral anticoagulants is associated with their bleeding tendency in standard clinical setup [58,59,60], which stresses the requirement for suitable use as per the product labeling for minimizing such risk [5,6,7]. An important limitation of this review was the inability to perform a subgroup analysis for the patients that were taking new oral anticoagulants before total hip replacement surgery due to limited data (i.e., non-reporting of whether patients were using any of the new oral anticoagulants before the surgery in the included studies). A subgroup analysis of such patients is recommended for the future studies since RDBCTs using heparins were performed in times when only warfarin was available as oral anticoagulant, whereas RDBCTs using direct oral anticoagulants have been performed in times when several antiplatelets or anticoagulants were available as antithrombotic treatment of several diseases. The withdrawal of these drugs before total hip replacement surgery could induce a bias of bleeding rate after surgery. 

## 4. Materials and Methods

The present systematic review and meta-analysis were performed using a pre-determined protocol according to the Preferred Reporting Items for Systematic Reviews and Meta-analyses (PRISMA) guidelines [61]. The PICO (Population, Intervention, Comparator, Outcome) criteria were defined *a priori* (Table 4, as defined in the research protocol (CRD42021266102).

### 4.1. Data Sources 

An electronic search was conducted using Cochrane Library, Elsevier’s Scopus, Clarivate Analytics’ Web of Science, Embase, and PubMed/Medline search engines (from inception to 31 July 2021) together with the United States trial registry (www.ClinicalTrial.gov, accessed on 31 July 2021) for the identification of relevant studies. The search was restricted to RDBCTs that compared enoxaparin with any of the approved new oral anticoagulants (i.e., rivaroxaban, apixaban, and dabigatran) for preventing VTE in human patients undergoing THR. There was no restriction of publication language and year. The following MESH keywords/terms were utilized: “enoxaparin”, “rivaroxaban”, “apixaban”, “dabigatran”, “venous thromboembolism”, “pulmonary embolism”, “deep venous thrombosis”, “thromboprophylaxis”, “total hip replacement”, and “total hip arthroplasty”. Furthermore, the bibliographies of eligible studies were screened for additional articles. 

### 4.2. Study Selection 

The titles and abstracts of the articles were screened for evaluating the contents of potentially eligible articles. The eligibility criteria for inclusion were as follows:

- RDBCTs that reported the effectiveness and safety of enoxaparin (with approved doses of 40 mg/day initiated 12 h prior to surgery [Europe] or 30 mg twice/day initiated 12–24 h after surgery [North America]) for thromboprophylaxis in comparison to either of the new oral anticoagulants (rivaroxaban with an approved dose of 10 mg once/day, apixaban with an approved dose of 5 mg once/day, or dabigatran with an approved dose of 150 mg or 220 mg once/day);
- RDBCTs that included human patients of all ages undergoing THR;
- RDBCTs that provided safety/efficacy information such as any DVT diagnosed by venography, hemorrhage, major bleeding, or pulmonary embolism (as defined by the authors);
- All other studies were excluded.

### 4.3. Data Extraction 

The following information was extracted from the included studies: (1) title of the article; (2) journal of publication; (3) total number of patients included in the study; (4) intervention including enoxaparin and the comparator drug; (5) duration of the treatment [years]; (6) follow-up duration [days]; (7) duration of surgery [min]; (8) percentage of females; (9) mean age [years] and weight [kgs]; (10) BMI [kg/m^2^]; (11) number of subjects for primary efficacy analysis; and (12) main findings of the study. 

### 4.4. Assessed Outcomes

The primary efficacy outcome of this systematic review was the combination of DVT, non-fatal pulmonary embolism, and death due to any reason during treatment. Most of the included studies also reported secondary efficacy outcomes. In the present review, major VTE was the secondary efficacy outcome, which was defined as the amalgamation of proximal DVT, non-fatal pulmonary embolism, and VTE-associated death. 

The primary safety outcome of this systematic review was bleeding events including minor events, clinically significant non-fatal bleeding events, or major bleeding events. A major bleeding event was defined as the one that led to the transfusion of ≥2 units of blood or clinically overt bleeding related with a reduction of hemoglobin level of a minimum of 2 g/dL, or that mandated supplementary surgeries, or that took place in a critical organ, or that was fatal. 

### 4.5. Assessment of Methodological Quality 

The potential risk of bias in the included studies was evaluated utilizing the Cochrane Collaboration’s risk of bias tool [62], which evaluates the following seven probable risks of bias: (a) random sequence generation; (b) allocation concealment; (c) blinding of subjects and personnel; (d) blinding of outcome assessor; (e) incomplete outcome data; (f) selective reporting; and (g) other sources of bias. 

### 4.6. Statistical Analysis 

Direct and indirect comparisons between enoxaparin versus apixaban, rivaroxaban, and dabigatran were carried out as per the recommendations of PRISMA [61]. For conducting the meta-analysis, relative risks (RR) and their corresponding 95% confidence intervals (CI) for individual publication were recorded. The Higgins I^2^ test [63] and the Cochran Q test [64] were used to assess the heterogeneity. A Cochran’s Q I^2^ > 50% and *p* < 0.10 were used for showing significant heterogeneity [65]. For the primary analysis, the random-effects model was used. The level of statistical significance was set as *p* < 0.05. The abovementioned analyses were carried out using a statistical software program (Stata, version 14.2; StataCorp LLC, College Station, TX, USA). Direct comparisons were performed utilizing the RevMan statistical software, version 5.1 (Nordic Cochrane Center, Copenhagen, Denmark) [66], while indirect comparisons (Bucher’s method) were performed using the Indirect Treatment Comparison (ITC), version 1.0 [67]. 

## 5. Conclusions

The findings of this meta-analysis revealed no significant difference in the efficacy and safety of new oral anticoagulants as compared to enoxaparin for the prevention of venous thromboembolism after total hip replacement surgery. 

## Figures and Tables

**Table 1:** Main characteristics of the included studies.

			Intervention										
**Study**	**Journal**	**No of Patients**	**Enoxaparin**	**Comparator**				**Duration of Treatment**	**Main Findings**				
Eriksson et al. (2006) [21]	*J. Thromb. Haemost.*	706	40 mg/day	2.5, 5, 10, 20, or 30 mg twice daily				5–9 days	When efficacy and safety were considered together, rivaroxaban at 2.5–10 mg b.i.d., compared favorably with enoxaparin for the prevention of VTE in patients undergoing elective THR.				
Eriksson et al. (2006) [22]	*Circulation*	873	40 mg/day	5, 10, 20, 30, or 40 mg twice daily				5–9 days	Rivaroxaban showed efficacy and safety similar to enoxaparin for thromboprophylaxis after THR, with the convenience of once-daily oral dosing and without the need for coagulation monitoring.				
Eriksson et al. (2007) [23]	*Thromb. Res.*	625	40 mg/day	2.5, 5, 10, 20 and 30 mg twice daily or 30 mg/day				5–9 days	This study demonstrated proof-of-principle for rivaroxaban for the prevention of VTE after THR.				
Eriksson et al. (2007) [24]	*Lancet*	3494	40 mg/day	150 or 220 mg/daily				28–35 days	Oral dabigatran was as effective as enoxaparin in reducing the risk of VTE after THR, with a similar safety profile.				
Eriksson et al. (2008) [25]	*N. Engl. J. Med.*	4541	40 mg/day	10 mg/day				31–39 days	A once-daily, 10-mg oral dose of rivaroxaban was significantly more effective for extended thromboprophylaxis than a once-daily, 40-mg subcutaneous dose of enoxaparin in patients undergoing THR. Moreover, the two drugs had similar safety profiles.				
Kakkar et al. (2008) [26]	*Lancet*	2509	40 mg/day	10 mg/day				Enoxaparin: 10–14 days Rivaroxaban: 31–39 days	Extended thromboprophylaxis with rivaroxaban was significantly more effective than short-term enoxaparin plus placebo for the prevention of VTE, including symptomatic events, in patients undergoing THR.				
Lassen et al. (2010) [27]	*N. Engl. J. Med.*	5407	40 mg/day	2.5 mg twice daily				35 days	Among patients undergoing THR, thromboprophylaxis with apixaban, as compared with enoxaparin, was associated with lower rates of VTE, without increased bleeding.				
Eriksson et al. (2011) [28]	*Thromb. Haemost.*	2055	40 mg/day	220 mg/day				28–35 days	Extended prophylaxis with oral dabigatran 220 mg once-daily was as effective as subcutaneous enoxaparin 40 mg once-daily in reducing the risk of VTE after THR, and superior to enoxaparin for reducing the risk of major VTE. Moreover, the risk of bleeding and safety profiles were similar.				
Eriksson et al. (2015) [29]	*Thromb. J.*	4374	40 mg/day	220 mg/day				28–35 days	Extended prophylaxis with oral dabigatran 220 mg once daily was as effective as enoxaparin 40 mg once daily in reducing the risk of total VTE and all-cause mortality after THR, with a similar bleeding profile. The clinically relevant outcome of major VTE and VTE-related death was significantly reduced with dabigatran versus enoxaparin.				
**Study**				**Follow-Up (days)**		**Duration of Surgery (min)**			**Females (%)**	**Mean Age (Years)**	**Mean Weight (kgs)**	**BMI (kg/m^2^)**	**No of Subjects for Primary Efficacy Analysis**
Eriksson et al. (2006) [21]				30–60		85.6			59	65.3	77.5	28	548
Eriksson et al. (2006) [22]				30–60		86.8			58.6	64.9	76.4	27.2	618
Eriksson et al. (2007) [23]				30–60		-			59	65	78.5	28	55
Eriksson et al. (2007) [24]				94		85			56	64	79	N/A	2651
Eriksson et al. (2008) [25]				30–35		91			55.5	63.2	78.2	28	1492
Kakkar et al. (2008) [26]				30–35		94			53.6	61.5	74.7	26.9	864
Lassen et al. (2010) [27]				65–95		88			52.8	60.5	79.6	28.1	2029
Eriksson et al. (2011) [28]				90		80			51.8	62	79	27.8	1577
Eriksson et al. (2015) [29]				90		85			54	63	79	27.8	243
**Study**				**Death**	**Major, Postsurgical Bleeding**		**Critical/Fatal Bleeding**		**Clinically Overt Bleeding Associated with Fall in Hb of ≥2 g/dL**		**Clinically Overt Bleeding Leading to Transfusion of ≥2 Units of Blood**		**Clinically Overt Bleeding Leading to Re-Operation**
Eriksson et al. (2006) [21]				-	17		0		8		11		4
Eriksson et al. (2006) [22]				0	27		0		18		24		2
Eriksson et al. (2007) [23]				2	21		-		17		13		2
Eriksson et al. (2007) [24]				6	56		2		42		45		8
Eriksson et al. (2008) [25]				9	8		1		3		3		3
Kakkar et al. (2008) [26]				10	2		0		1		1		0
Lassen et al. (2010) [27]				7	40		0		23		30		2
Eriksson et al. (2011) [28]				2	23		0		20		18		0
Eriksson et al. (2015) [29]				4	64		1		50		55		5

**Abbreviations:** BMI = body mass index; THR = total hip replacement; VTE = venous thromboembolism.

**Table 2:** Indirect comparisons between apixaban, rivaroxaban, and dabigatran *.

	Relative Risk (95% CI)		
Outcomes	Rivaroxaban vs. Dabigatran	Rivaroxaban vs. Apixaban	Apixaban vs. Dabigatran
Symptomatic venous thromboembolism	0.76 (0.29 to 2.10)	0.61 (0.29 to 1.22)	1.20 (0.34 to 4.16)
Clinically relevant bleeding	1.24 (0.95 to 1.53)	1.49 (1.27 to 1.74)	0.77 (0.59 to 0.88)
Major bleeding	1.65 (0.87 to 2.53)	1.63 (0.80 to 2.98)	0.83 (0.39 to 1.76)
Net clinical endpoint	0.89 (0.69 to 1.56)	0.98 (0.72 to 1.38)	0.99 (0.72 to 1.71)

* Random effects model, events while receiving treatment.

**Table 3:** Direct and indirect comparisons: absolute difference in events per 1000 patients treated *.

	Risk Difference (95% CI)			
Comparison	Symptomatic Venous Thromboembolism	Clinically Relevant Bleeding	Major Bleeding	Net Clinical Endpoint
**Direct comparisons:**				
*Enoxaparin* vs. *Rivaroxaban*	−4 (−8 to −1)	8 (3 to 18)	4 (−0.6 to 9)	−4 (−8 to 3)
*Enoxaparin* vs. *Dabigatran*	−2 (−8 to 4)	4 (−3 to 12)	−1 (−5 to 4)	−1 (−7 to 8)
*Enoxaparin* vs. *Apixaban*	−1 (−5 to 2)	−7 (−14 to −1)	−1 (−6 to 4)	−1 (−5 to 2)
**Indirect comparisons:**				
*Rivaroxaban* vs. *Dabigatran*	−3 (−10 to 3)	4 (−8 to 15)	4 (−3 to 12)	−2 (−11 to 8)
*Rivaroxaban* vs. *Apixaban*	−4 (−8 to 2)	17 (8 to 29)	4 (−3 to 11)	−2 (−8 to 5)
*Dabigatran* vs. *Apixaban*	1 (−8 to 7)	−12 (−23 to −3)	0 (−7 to 6)	0 (−8 to 8)

* Random effects model, events while receiving treatment.

**Table 4:** PICO criteria.

Population (P)	Subjects who have undergone total hip replacement surgery
Intervention (I)	Enoxaparin (low-molecular-weight heparin) Comparator (i.e., Apixaban, Rivaroxaban and/or dabigatran)
Comparator (C)	Efficacy and safety outcomes of enoxaparin will be compared with apixaban, rivaroxaban, and/or dabigatran
Outcomes (O)	*Primary efficacy outcomes:* a combination of DVT, non-fatal pulmonary embolism, and death due to any reason during treatment. *Secondary efficacy outcomes:* major venous thromboembolism (combination of proximal DVT, non-fatal pulmonary embolism, and VTE-associated death). *Safety outcomes:* bleeding events including minor events, clinically significant non-fatal bleeding events, or major bleeding events.

## Data Availability

Not available.
